# Supplementary material for: A CRISPR-engineered isogenic model of the 22q11.2 A-B syndromic deletion
Source: Sci Rep. 2023 May 11;13:7689. doi: 10.1038/s41598-023-34325-2 (PMC10175260; doi:10.1038/s41598-023-34325-2)
Supplement: Supplementary file 1 — Supplementary Information. [file 41598_2023_34325_MOESM1_ESM.pdf]

SUPPLEMENTARY FIGURE 1

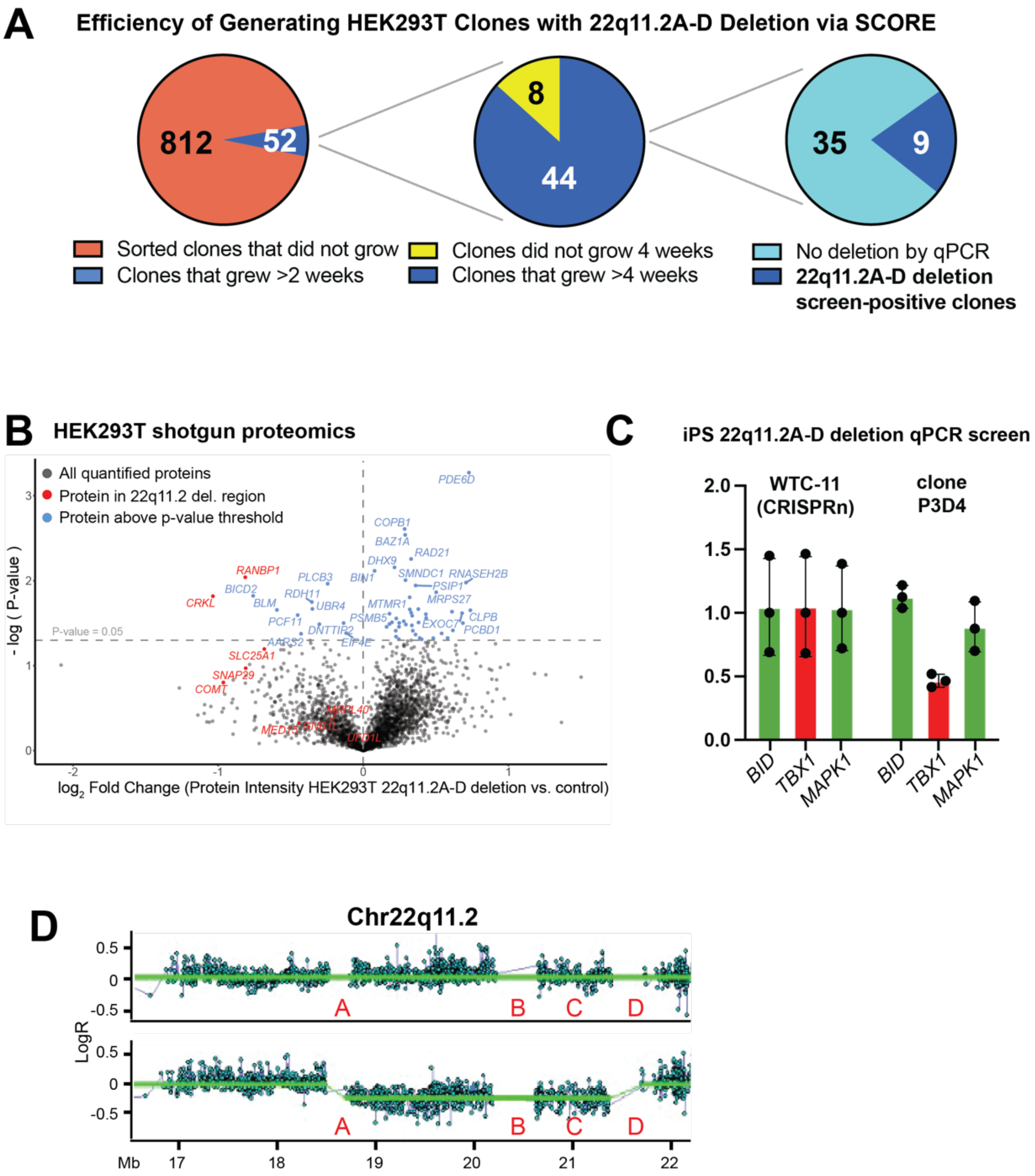

Supplementary Figure 1. CRISPR engineering of 22q11.2 A-D deletion in HEK293T cells and iPS cells

A. Single cell sorting and clonal expansion of HEK cells yielded 9 deletion harboring clones from 44 total clones as determined by qPCR.

B. Volcano plot comparing LFQ intensity of proteins in 22q11.2 deletion clones vs control clones (n = 3 clones each).

C. qPCR of a single 22q11.2 A-D deletion clone (clone P3D4) showing reduction in copy number of gene *TBX1* within the deletion in comparison to genes (*BID* and *MAPK1*) flanking the

deleted region. Control iPS cells (CRISPRn/WTC-11) are shown for comparison. *n* = 3 technical replicates, +/- S.D. shown.

D. SNP microarray data of the same screen-positive deletion iPS clone confirms the presence of the 22q11.2 A-D deletion (bottom plot). CRISPRn/WTC-11 data shown for comparison (top plot).

SUPPLEMENTARY FIGURE 2

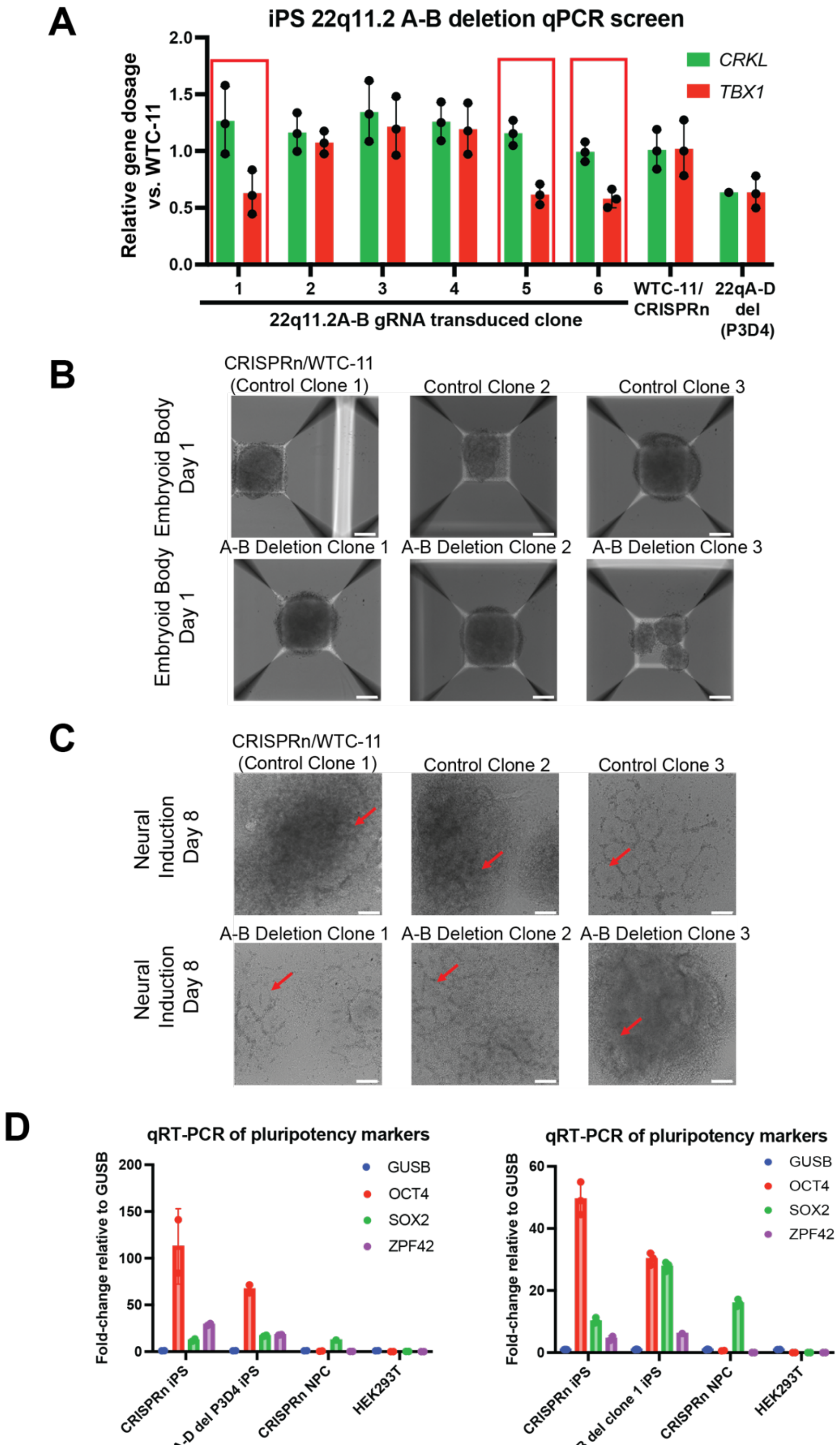

Supplementary Figure 2. Characterizing 22q11.2 A-B deletion iPS lines.

A. qPCR of a subset of screened 22q11.2 A-B gRNA transduced clones, showing reduction in copy number of a gene (*TBX1*) within the deletion in comparison to a gene (*CRKL*) flanking the A-B deleted region (*CRKL* located within the C-D region, see Fig. 1A). Control iPS cells and single identified clone with A-D deletion (clone P3D4) are shown for comparison. Red boxes indicate screen-positive clones (see Fig. 2B). *n* = 3 technical replicates, +/- S.D. shown.
B. Representative microscopy images of control and deletion iPSC differentiation into embryoid bodies within Aggrewell™ plates. 10x magnification. Scale bar = 100 mm.
C. Representative microscopy images of control and deletion iPSC differentiation into neuronal progenitor cells (NPCs) via formation of neural rosettes (individual rosettes highlighted by red arrows). 10x magnification. Scale bar = 100 mm.
D. Characterization of 22q11.2 A-D (*left*) and A-B deletion iPS (*right*) clones by qRT-PCR of standard pluripotency markers. Comparison is shown to WT CRISPRn/WTC-11 iPS and NPC clones, as well as HEK293T cells (*n* = 2-3 technical replicates per marker).

Supplementary Table 1. CNVs identified by SNP array in iPS clones

iPSC line***	Description	22q11.2 deletion (size)	Other pathogenic* CNVs. (size)	Other VUS >500 kb** (size)
CRISPRn/WTC-11	Parental iPS line harboring dox-inducible Cas9 (control clone 1)	No	No	No
22qAB_g3_B1_5	22q11 A-B deletion clone 1	Yes (1.413 Mb)	No	No
22qAB_g3_B1_6	22q11 A-B deletion clone 2	Yes (1.413 Mb)	No	No
22qAB g3_B2-4	22q11 A-B deletion clone 3	Yes (1.399 Mb)	No	No
22qAB_g3_B2_1	Control clone 2	No	No	No
22qAB_g3_B2_3	Control clone 3	No	No	No
22qAB_g3_B1_1	22q11 A-B deletion, not used in further study	Yes (1.413 Mb)	1q24.2q41 copy gain (52.454 Mb)	No
22qAB_g3_B2_2	22q11 A-B deletion, not used in further study	Yes (1.412 Mb)	1q24.2q41 copy gain (52.477 Mb)	No

CNV = copy number variant
VUS = variant of uncertain significance, as defined by reporting criteria of Kearney et al. *Genet Med* **13**:680 (2011).
*Pathogenic CNVs are defined using reporting criteria of Kearney et al. (2011).
We note that the CRISRPn/WTC-11 line (Miyaoka et al. *Nat Methods* **11:291 (2013)) is engineered from the parental WTC-11 line, derived from a phenotypically normal male donor

(karyotype 46,XY) (Coriell, GM25256). We found that CRISPRn/WTC-11 harbors two variants of uncertain significance detected by SNP array: a Yp11.2 copy loss of 2800 kb (ISCN nomenclature Yp11.2(6370460_9170545)x0) and a 7q11.22 copy loss of 877 kb (ISCN nomenclature 7q11.2(69298131_70158495)x1). These copy losses are present in all clones presented here. This column reflects the presence of any additional copy number changes of uncertain significance >500 kb in size, not present in the parental CRISPRn/WTC-11 line.

***All clones except CRISPRn/WTC-11 were single-cell sorted from a bulk population that were transfected with the sgRNA designed to generate the A-B deletion and had Cas9 induced by doxycycline (see Methods). CRISPRn/WTC-11 parental iPS cells used as a control were single cell sorted from a bulk population of these cells, which were not transfected with sgRNA.

Supplementary Table 2. List of genes positively and negatively correlated with neuron maturation or proliferation as identified in GSEA.

GSEA for Transcriptional Signatures of Neuron Maturation						
NAME*	SYMBOL**	TITLE	RANK IN GENE LIST	RANK METRIC SCORE	RUNNING ES***	CORE ENRICHMENT
row_0	KCNB1	NA	107	1.575709	0.046156	Yes
row_1	ACTL6B	NA	202	1.439835	0.088532	Yes
row_2	ADGRB3	NA	238	1.404893	0.132895	Yes
row_3	ANKS1A	NA	249	1.395437	0.178276	Yes
row_4	SRRM4	NA	384	1.298025	0.213861	Yes
row_5	MAP3K13	NA	468	1.255069	0.250743	Yes
row_6	GLDN	NA	677	1.160527	0.277869	Yes
row_7	LGI4	NA	812	1.113008	0.307366	Yes
row_8	BCL11A	NA	949	1.070315	0.335352	Yes
row_9	MECP2	NA	1016	1.053457	0.366504	Yes
row_10	RND1	NA	1312	0.978772	0.383026	Yes
row_11	KDM1A	NA	2220	0.815072	0.36163	Yes
row_12	CNTNAP2	NA	2391	0.786563	0.378472	Yes
row_13	RAC3	NA	2583	0.755325	0.393171	Yes
row_14	KCNIP2	NA	2673	0.741597	0.41284	Yes
row_15	NR4A2	NA	2705	0.737109	0.435444	Yes
row_16	RET	NA	3041	0.679689	0.439999	Yes
row_17	SPTBN4	NA	3141	0.665731	0.456641	Yes
row_18	SCARF1	NA	3463	0.61303	0.459747	Yes
row_19	NTN4	NA	3764	0.571551	0.462605	Yes
row_20	PICK1	NA	3971	0.541508	0.469471	Yes
row_21	APP	NA	4561	0.471724	0.453682	Yes
row_22	EPHA8	NA	4644	0.461866	0.464519	Yes
row_23	B4GALT6	NA	4767	0.443475	0.472625	Yes
row_24	C1QA	NA	5125	0.423611	0.467585	No
row_25	NRCAM	NA	5921	0.327533	0.436101	No
row_26	CX3CL1	NA	6656	0.248132	0.405248	No
row_27	FEV	NA	6722	0.23823	0.409631	No
row_28	MYOC	NA	7016	0.202936	0.400732	No
row_29	VSX1	NA	7267	0.171168	0.393075	No
row_30	TBX6	NA	7710	0.123862	0.373654	No
row_31	B4GALT5	NA	8337	0.048681	0.34198	No
row_32	CNTN2	NA	9926	-0.06719	0.259776	No
row_33	MTCH1	NA	10301	-0.10999	0.243514	No
row_34	SCLT1	NA	11383	-0.23608	0.193818	No
row_35	BCL2	NA	11406	-0.23836	0.200491	No
row_36	AGRN	NA	11855	-0.29074	0.186243	No

row_37	FARP2	NA	13273	-0.43322	0.125172	No
row_38	C3	NA	13626	-0.47176	0.121982	No
row_39	EDNRB	NA	13867	-0.5012	0.125715	No
row_40	LRRK2	NA	14379	-0.55779	0.116904	No
row_41	EDNRA	NA	15239	-0.64594	0.092494	No
row_42	C1QL1	NA	15778	-0.70792	0.087187	No
row_43	RB1	NA	17061	-0.87655	0.047879	No
row_44	CLN5	NA	18599	-1.44325	0.013662	No
**GSEA for Transcriptional Signatures of Neuronal Proliferation Markers**						
NAME*	SYMBOL**	TITLE	RANK IN GENE LIST	RANK METRIC SCORE	RUNNING ES***	CORE ENRICHMENT
row_0	SOX10	NA	78	1.63939	0.048603	No
row_1	TOX	NA	1590	0.915563	-0.0023	No
row_2	SMARCD3	NA	1988	0.849909	0.003934	No
row_3	LHX1	NA	2022	0.843859	0.029332	No
row_4	DISP3	NA	2120	0.832402	0.050958	No
row_5	KDM1A	NA	2220	0.815072	0.07192	No
row_6	DLL4	NA	2667	0.743229	0.072114	No
row_7	LHX5	NA	2842	0.711141	0.085743	No
row_8	ID4	NA	3367	0.627537	0.078066	No
row_9	CX3CR1	NA	4654	0.461063	0.024505	No
row_10	DMRTA2	NA	5251	0.413351	0.006107	No
row_11	GJC2	NA	6057	0.310989	-0.0267	No
row_12	CX3CL1	NA	6656	0.248132	-0.05052	No
row_13	PAX6	NA	6948	0.210899	-0.05921	No
row_14	DRD2	NA	7231	0.175775	-0.06855	No
row_15	SHH	NA	7558	0.140075	-0.08139	No
row_16	GPR37L1	NA	7770	0.118356	-0.0888	No
row_17	INSM1	NA	8114	0.076761	-0.10457	No
row_18	ASCL1	NA	9398	-0.00691	-0.17259	No
row_19	ZNF335	NA	9557	-0.02135	-0.1803	No
row_20	FZD3	NA	9883	-0.06219	-0.19559	No
row_21	LRP2	NA	9896	-0.06372	-0.19418	No
row_22	LHX2	NA	10071	-0.08504	-0.20069	No
row_23	FZD9	NA	10600	-0.1425	-0.22419	No
row_24	DISC1	NA	10725	-0.16105	-0.2256	No
row_25	ADGRG1	NA	11228	-0.21644	-0.24534	No
row_26	PITX3	NA	11278	-0.223	-0.24077	No
row_27	OTP	NA	11337	-0.23079	-0.23643	No
row_28	RASSF10	NA	11417	-0.23951	-0.23292	No
row_29	CDON	NA	11419	-0.23965	-0.22526	No
row_30	PROX1	NA	11505	-0.25123	-0.2217	No

row_31	DCT	NA	12593	-0.38168	-0.26723	No
row_32	NR2E1	NA	12863	-0.41432	-0.26821	No
row_33	ASPM	NA	13276	-0.43356	-0.27617	No
row_34	EGF	NA	13412	-0.44987	-0.26887	No
row_35	HIF1A	NA	13792	-0.49149	-0.27322	No
row_36	VEGFA	NA	13851	-0.49864	-0.26026	No
row_37	NES	NA	14833	-0.60511	-0.29296	No
row_38	CTNNB1	NA	15292	-0.65175	-0.29635	Yes
row_39	GLI1	NA	15556	-0.6813	-0.28841	Yes
row_40	VEGFC	NA	15562	-0.68152	-0.26675	Yes
row_41	ELL3	NA	15818	-0.71348	-0.25735	Yes
row_42	WDR62	NA	15881	-0.72058	-0.23746	Yes
row_43	MDK	NA	16407	-0.78464	-0.24014	Yes
row_44	WNT3A	NA	16580	-0.811	-0.22319	Yes
row_45	NAP1L1	NA	16591	-0.8121	-0.19759	Yes
row_46	FGF2	NA	16684	-0.82525	-0.17593	Yes
row_47	NOTCH1	NA	17155	-0.89493	-0.17213	Yes
row_48	FOXG1	NA	17643	-0.98806	-0.16624	Yes
row_49	SMO	NA	17825	-1.03623	-0.14252	Yes
row_50	ITGB1	NA	17959	-1.07539	-0.11499	Yes
row_51	GLI3	NA	18005	-1.08978	-0.08232	Yes
row_52	LYN	NA	18353	-1.23258	-0.06111	Yes
row_53	GNG5	NA	18486	-1.31966	-0.02567	Yes
row_54	GNAI2	NA	18582	-1.40755	0.014573	Yes

* Name corresponds to the row number where the genes associated with a gene set fall within waterfall plot of GSEA shown in Figure 4D-E.

** Gene symbol of maturation/ proliferation genes within gene list used for GSEA to identify correlation of transcript data for 3-week deletion neurons with signatures of neuron maturation/ proliferation (Gene sets used for analysis include GO:0014042 Positive regulation of neuron maturation; GO:0014043 Negative regulation of neuron maturation; GO:2000179 Positive regulation of neural precursor cell proliferation; GO:2000178 Negative regulation of neural precursor cell proliferation)

***ES: Enrichment Score. The cumulative enrichment score (ES) is defined as the maximum deviation from the starting point. The ES in the maturation plot is 0.47 and, in the proliferation plot it is 0.32, suggesting that our deletion clones show positive transcriptional signatures for maturation and negative signatures of proliferation.

SUPPLEMENTARY FIGURE 3

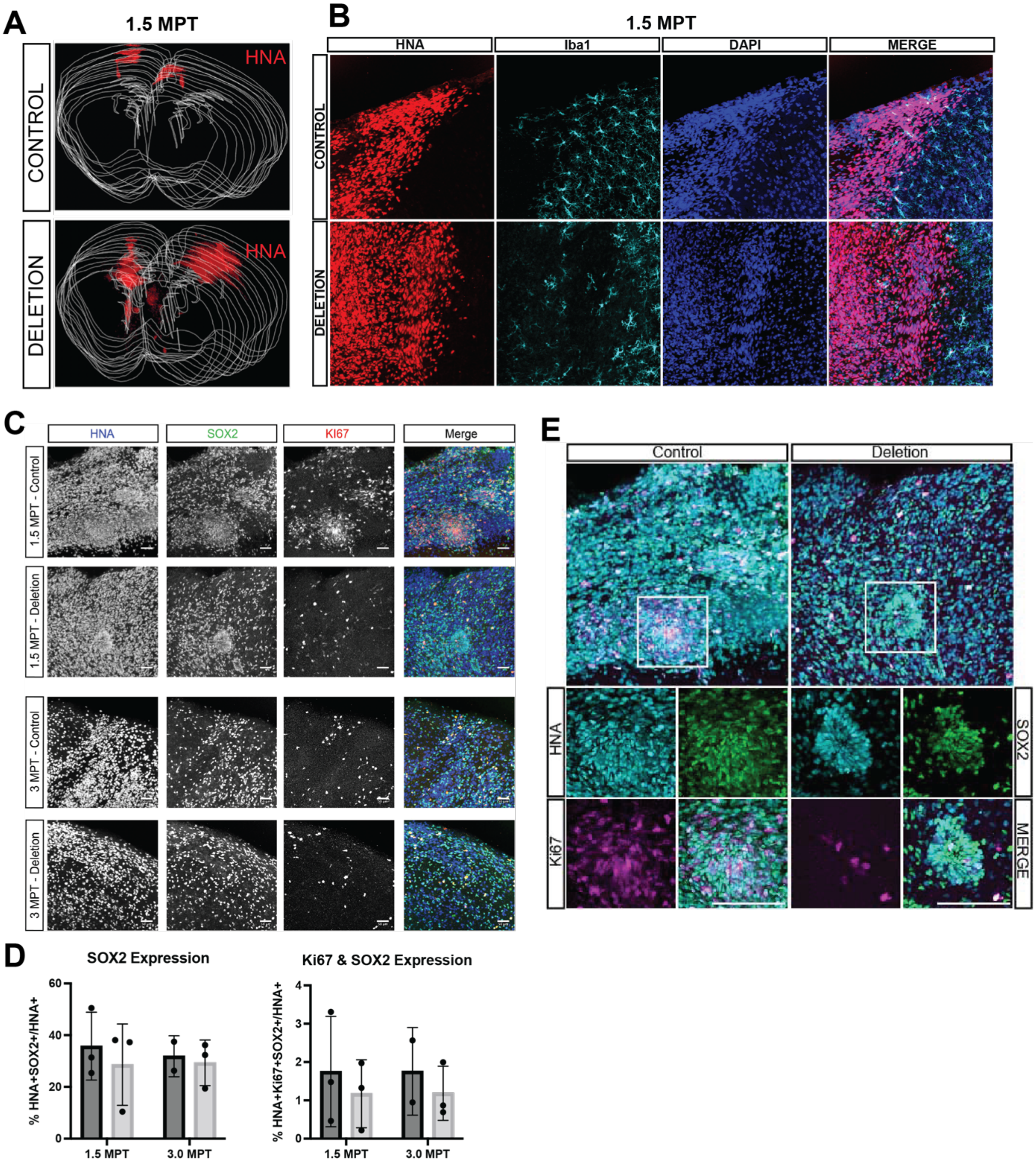

Supplementary Figure 3. Properties of xenotransplanted control versus 22Q11.2 A-B deletion neuronal progenitor cells.

A. Three-dimensional rendering of serial coronal sections of xenotransplanted mouse brains using control (top) or 22q11.2 deletion cell lines. Each red dot represents a xenotransplanted cell.

B. Confocal images of control cells or 22q11.2 cell lines (identified by HNA in red) at the transplant site and surround microglia, as identified by Iba1 (cyan). Cell nuclei identified by DAPI (blue). Morphology of Iba1+ cells appear non-reactive. Representative image shown from control clone 1 and deletion clone 3.
C. Transplanted HNA+ cells (blue) co-express both SOX2 (green) and KI67 (red) at 1.5 MPT (1st and 2nd rows) and 3 MPT (3rd and 4th rows) in control vs. 22q11.2 deletion xenografts. Representative images shown from control clone 3 and deletion clone 2.
D. (*Left*) Quantification of HNA+ cells that co-express SOX2 in control (dark gray) and 22q11.2 deletion (light gray) at 1.5 MPT and 3 MPT. (*Right*) Quantification of HNA/KI67/SOX2 triple-positive transplanted cells in control (dark gray) and 22q11.2 deletion (light gray) at 1.5 MPT and 3 MPT. Quantification performed across control clones 1-3 and deletion clones 1 and 3 at 1.5 MPT (each data point average of n = 3 mice per clone); control clones 2 and 3 and deletion clones 1 and 2 at 3 MPT (each data point average of n = 2 mice per clone).
E. Confocal images of injection sites containing neural rosettes formed at 1.5 MPT in control (1st row; left panel) and 22q11.2 deletion (1st row; right panel) contain transplanted cells that are triple-positive (white) for HNA+ (cyan), SOX2 (green) and KI67 (magenta). Higher magnification of neural rosettes (white boxes) show larger rosettes with more triple-positive cells present in control clone 3 (2nd and 3rd row; *left*) vs. 22q11.2 deletion clone 2 (2nd and 3rd row; *right*).
